# Central retinal vein occlusion secondary to severe iron-deficiency anaemia resulting from a plant-based diet and menorrhagia: a case presentation

**DOI:** 10.1186/s12886-020-01372-6

**Published:** 2020-03-19

**Authors:** Verlyn Yang, Liam Daniel Turner, Fraser Imrie

**Affiliations:** Ophthalmology Department, Gold Coast University Hospital, 1 Hospital Boulevard, Southport, Queensland 4215 Australia

**Keywords:** Central retina vein occlusion, Iron deficiency, Anaemia, Vegan, Vegetarian, Anti-VEGF

## Abstract

In this case presentation, we present a young vegan patient who developed a CRVO secondary to severe iron-deficiency anaemia (IDA) attributable to menstrual losses and limited iron intake. CRVO is a rare complication of IDA.

With rising calls for sustainable diets and rising evidence for a plant-based diet, there has been a rise in popularity of such diet forms. While there are ocular benefits from this diet trend, the potential for nutritional deficiencies including iron needs to be monitored especially in susceptible individuals. Iron is essential for retina metabolism and function; however, excess iron contributes to disease states in the eye. Therefore, supplementation needs to be judicious.

## Background

Central retinal vein occlusion (CRVO) is the second most common retinal vascular disease, following diabetic retinopathy [1].

Iron deficiency anaemia (IDA) is a known cause of CRVO, with previously documented cases in the literature [2–6]. The underlying mechanism of thrombosis in IDA is the result of: 1) reactive thrombocytosis (e.g. increased platelet count, and activity); 2) hypoxia from anaemia leading to injury of endothelial cells in the retino-choroidal circulation; and 3) dysregulation of coagulation (e.g. fibronlysis). Life-threatening complications of iron deficiency anaemia have also been reported with cases of cerebral vascular accidents, pulmonary thromboembolism, and cerebral venous thrombosis also arising [7–9].

Iron is essential in retina metabolism but a state of haemostasis needs to exist as high levels of iron contribute to a range of disorders.

Plant-based diets have gained widespread popularity globally in the last decade, with reports of 70% of the world population actively reducing meat consumption, a trend backed by the millennials [10]. In Australia, 11.2% of the population have identified with veganism in 2016, up from 9.7% in 2014 [11]. Similar trends have been observed in America, United Kingdom, Germany and Asia.

In this case presentation, we present a young vegan patient who developed a CRVO secondary to severe IDA attributable to menstrual losses and limited iron intake.

## Case presentation

A 21-year-old female presented with a 6-day duration of reduced right eye vision on a background of 6 weeks of low-grade headaches and associated fatigue. Her past medical history was remarkable for dysmenorrhea, which included cycles of menorrhagia interspersed with amenorrhea, and a plant-based diet. Relevant negatives included no previous miscarriages, venous thrombo-embolism, diabetes mellitus, hypertension or raised intra-ocular pressures.

On general examination, she had was normotensive and had a normal blood sugar level. Ophthalmologic exam showed unaided best corrected visual acuity (BCVA) of hand motion in the right eye and 6/4 in the left eye with no improvement on pin-hole. Intraocular pressures were normal at 12 mmHg in the right, and 16 mmHg in the left. Slit lamp exam of the anterior segments were within normal, with rubeosis excluded.

**Fig. 1:**
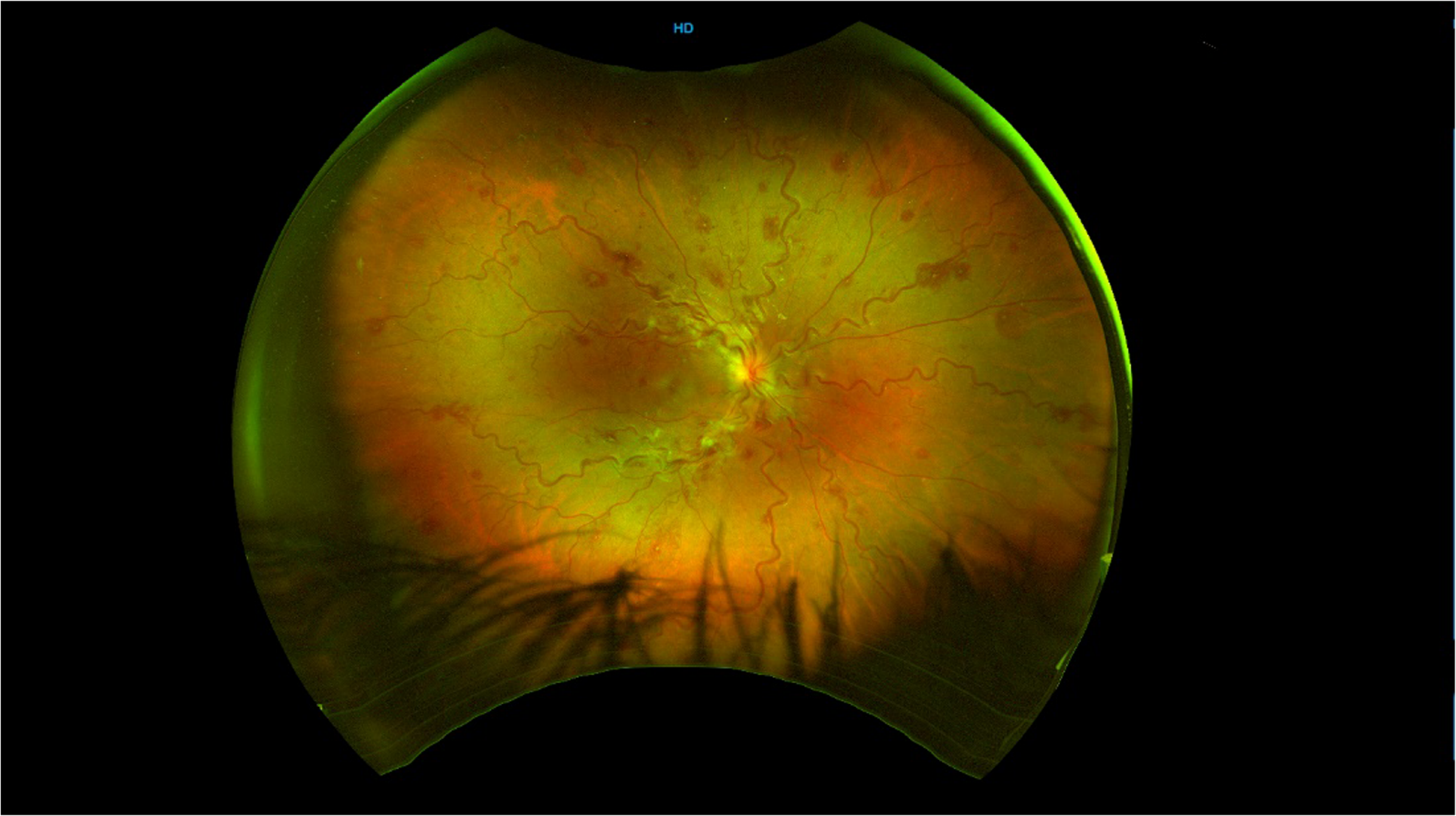
Colour fundus photograph showing tortuosity of vessels, dot and blot haemorrhages, cotton wool spots and optic disc swelling

**Fig. 2:**
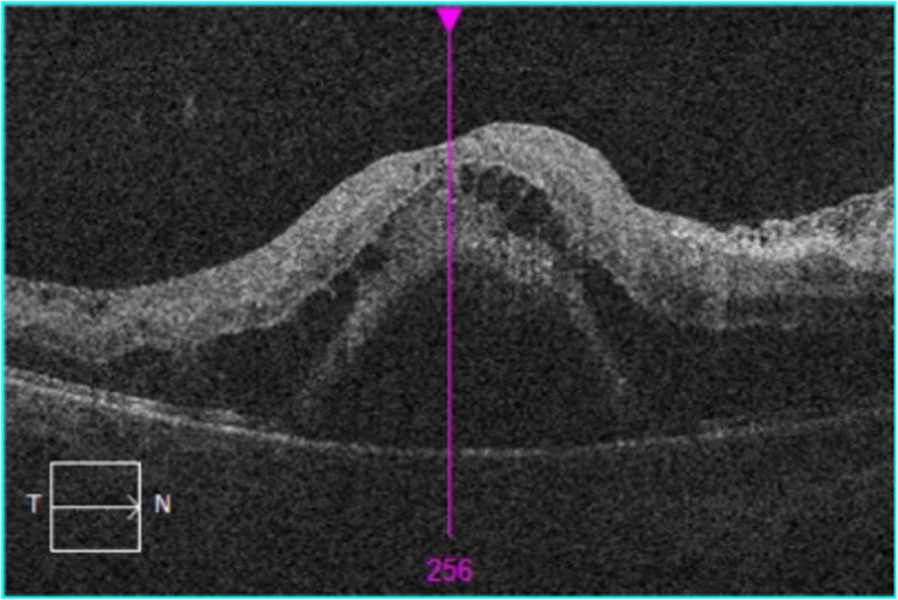
OCT Findings showing large amounts of CMO and sub-retinal fluid

Fundus examination of the right eye revealed a swollen optic nerve head, intraretinal hemorrhages in all 4 quadrants, tortuosity of the retinal veins, cotton wool spots and associated cystoid macular oedema (CMO) (Figs. 1 and 2). There were no signs of neovascularization of the disc or fundus. Fundus examination of the left eye was normal with a healthy optic disc head and macula. The patient was diagnosed with a right eye CRVO.

An extensive work-up was undertaken. There was a profound microcytic anaemia with haemoglobin of 40 g/L (reference range 115–160) and Mean Corpuscular Volume of 68 fL (reference range 80–100).. Platelets were normal at 362 × 10^ [9]/L (reference range 140–400 × 10^ [9]/L). The iron studies pointed conclusively to an iron deficiency with iron levels < 2 umol/L (reference range 9–30), and low iron stores with high transferrin levels of 3.9 g/L (reference range 2.0–3.6) and low transferrin saturations < 2% (reference range 15–45).

All other laboratory parameters, were normal including: white cell count, electrolytes, urea, liver function, beta-hCG, coagulation prolife, thrombophilia screen, anti-nuclear antibodies, anti-cardiolipin antibodies, anti-beta 2 glycoprotein, tuberculosis, syphilis, hepatitis, human immunodeficiency virus and vitamin B12 and folate. Neuro-imaging in the form of MRI was undertaken to rule out a vasculitis and cerebral venous thrombosis which returned negative.

The profound anaemic status and iron-deficiency was largely attributed to the lack of iron intake through her plant-based diet, with a contributory role from her dysmenorrhea. The patient was treated with packed blood cell transfusion, intravenous iron supplementation and subsequent oral supplementation. She also received an intra-vitreal injection (IVI) of, an anti-vascular endothelial growth factor (VEGF), aflibercept.

**Fig. 3:**
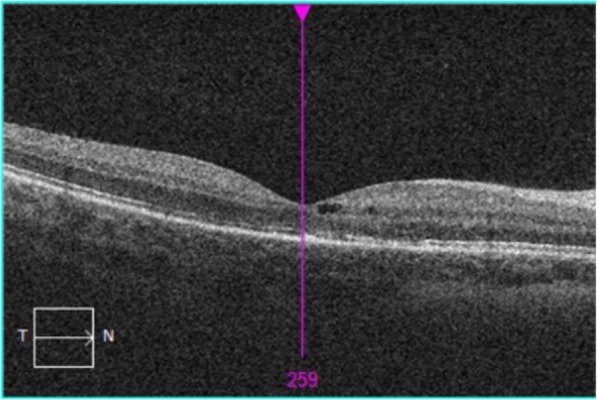
OCT 3 weeks after 1 treatment of intra-vitreal afibercept injection showing good response with mild CMO remaining, and resolution of sub-retinal fluid

Three weeks following systemic treatment and administration of aflibercept, the patient’s unaided BCVA was 6/9 in the affected eye. Fundus examination of the right eye revealed nearly complete resolution of CMO, dilatation of vessels, a residual optic disc swelling and cotton wool spots as previously noted. There were no signs of neovascularization in the eye. Fundus examination of the left eye was normal. OCT images were obtained (Fig. 3). A repeat IVI afibercept was administered at 4 weeks. With oral iron supplementation her haemoglobin returned to 121 g/L 6 weeks later.

Twelve months later, the patient’s unaided BCVA returned to 6/6 in the affected eye and there was full resolution of the CMO. She now incorporates some red meat into her plant-rich diet and has periodic blood tests to monitor for anaemia with her general practitioner.

## Discussion and conclusions

CRVO secondary to IDA has previously been reported in the literature [2–6, 12], but this is the first case with use of anti-VEGF in such a setting.

A variety of underlying mechanisms have been hypothesized to explain the thrombotic events in IDA including a direct process of reactive thrombocytosis, and other mechanisms following the principles of Virchow’s triad mainly endothelial injury and hypercoagulability. Iron functions to regulate platelet numbers and function by inhibiting thrombopoiesis [8]. In a state of iron-deficiency, there is a reactive thrombocytosis, thus leading to hypercoagulability. Red cell deformability is reduced in microcytic iron-deficient cells, resulting in an increased viscosity and furthermore contributing to the hypercoagulable state [13]. It has been hypothesized that anemic hypoxic injury to the retino-choroidal circulation causes endothelial cell dysfunction [14] and a weaker anti-oxidant defence in the IDA state, results in increased platelet aggregation [15].

Visual loss from CRVO is commonly secondary to macular oedema and treatment with IVI anti-VEGF with strong evidence backing the practice [16]. However, from our literature review, this is the first instance that anti-VEGF has been used in the context of macular oedema secondary to IDA-induced CRVO .

Dietary and lifestyle factors can affect the retina health and this has investigated in age-related macular degeneration (AMD) studies. The AREDS and AREDS2 studies found a supplementation of lutein and zeaxanthin to be helpful in reducing the risk of developing advanced AMD in the high-risk group [17, 18]. Lutein and zeaxanthin are carotenoids abundant in dark leafy vegetables. Further studies have found that people with high intake of vegetables had a lower risk for AMD compared to those with low intakes [19]. Studies have also shown the converse of high meat intake increasing the risk of AMD [20].

Iron is essential for retinal metabolism and the phototransduction. RPE-65 is an enzyme required to catalyse the conversion of all-trans-retinyl ester to 11-cis-retinol, a critical step in the visual cycle [21]. Photoreceptor regeneration also depends on iron-containing enzymes like fatty-acid desaturase for the synthesis of lipids required in the membranes [22].

Dietary iron can be obtained from both plant and animal sources. Theoretically, it is possible for one to obtain sufficient iron solely from plant-based products. However, this is often not the case and is related to the lower bioavailability of iron in plant products compared to animal sources [23, 24].

IDA is a common form of anaemia, affecting 4.5 to 18% of the US population, and rates of 64.7% reported in central Asia [25]. It is a disease of either insufficient dietary intake or absorption or excessive loss from bleeding – where both factors were present in our patient. Iron supplementation is appropriate in the setting of deficiency, however, should be judiciously administered. Excess iron in the form of ferrous can catalyze the conversion of hydrogen peroxide to highly reactive oxygen species (ROS) and radicals [26] with the products implicated in neurodegenerative diseases (e.g. Alzheimer’s disease) [27] and a range of ocular diseases (e.g. AMD, diabetic retinopathy and others) [22].

CRVO is an uncommon entity in the young adult population and a thorough search for an underlying systemic etiology must be undertaken. This case report illustrates a health-conscious young adult suffering from iron-deficiency anaemia secondary to a plant-based diet, and subsequently presenting with CRVO. A diet rich in vegetables is beneficial to retina health but a diet limited to plants-only predisposes one to potential nutritional deficiencies. Supplementation, while appropriate in deficiency states, needs to be judicious. The case highlights that individuals with vegan diets should have their hematocrits, serum iron levels, and serum B12 levels checked periodically, and given supplements if medically indicated.

## Data Availability

The data and material generated from the study can be obtained from the corresponding author, Dr. Verlyn Yang, at yangverlyn@gmail.com.

## Abbreviations

AMD: Age related macular degeneration
BCVA: Best corrected visual acuity
CRVO: Central retinal vein occlusion
CMO: Cystoid macular oedema
hCG: Human chorionic gonadotropin
IDA: Iron deficiency anaemia
IVI: Intra-vitreal injection
OCT: Optical coherence tomography
ROS: Reactive oxygen species
VEGF: Vascular endothelial growth factor
